# The liver in diabetes: current state and future perspectives

**DOI:** 10.1007/s00125-026-06827-x

**Published:** 2026-08-15

**Authors:** Hannele Yki-Järvinen, Michael Roden

**Affiliations:** 1 Department of Medicine, Helsinki University Hospital, University of Helsinki, Helsinki, Finland; 2 Minerva Foundation Institute for Medical Research, Helsinki, Finland; 3 Department of Endocrinology and Diabetology, Medical Faculty and University Hospital Düsseldorf, Heinrich Heine University, Düsseldorf, Germany; 4 Institute for Clinical Diabetology, German Diabetes Center, Leibniz Center for Diabetes Research at Heinrich Heine University Düsseldorf, Düsseldorf, Germany; 5 German Center for Diabetes Research (DZD), Partner Düsseldorf, München-Neuherberg, Germany

**Keywords:** Fatty liver, Insulin resistance, Liver fibrosis, Liver screening, MASH, MASLD, Metabolic dysfunction-associated steatotic liver disease, Metabolic syndrome, Novel MASH treatment, Review

## Abstract

Diabetes mellitus is strongly associated with metabolic dysfunction-associated steatotic liver disease (MASLD). For many years, MASLD has not been recognised as either a common pathophysiological feature of type 2 diabetes or a diabetes-related complication. This special issue of *Diabetologia* puts the liver in focus in the context of diabetes. This review discusses current challenges in, and emerging opportunities for, the diagnosis and management of MASLD and fibrosis in type 2 diabetes. Up to 95% of cases of MASLD remain undiagnosed because there are no accepted recommendations for assessing steatosis, whereas non-invasive tests overestimate the prevalence of MASLD progression in type 2 diabetes. Of note, the combination of type 2 diabetes and metabolic syndrome features is a strong predictor of liver disease outcomes, but CVD is the major cause of death in non-cirrhotic MASLD. MASLD is heterogeneous including subtypes linked to common genetic variants, for example *PNPLA3* I148M, which increases the risk of liver disease but may protect against CVD. Recent approval of drugs targeting hepatic energy metabolism or promoting weight loss is promising for achieving fibrosis remission, but people with type 2 diabetes will still require complex multifactorial management. Achieving a significant increase in diagnostic rates of at-risk metabolic dysfunction-associated steatohepatitis (MASH) will require a paradigm shift, including greater awareness among clinicians, including diabetologists and endocrinologists, and expansion of community-based diagnostic capabilities accompanied by analyses of the cost-effectiveness of prevention and treatment.

**Supplementary Information:**

The online version contains a slideset of the figures for download available at https://doi.org/10.1007/s00125-026-06827-x.

**Figure:**
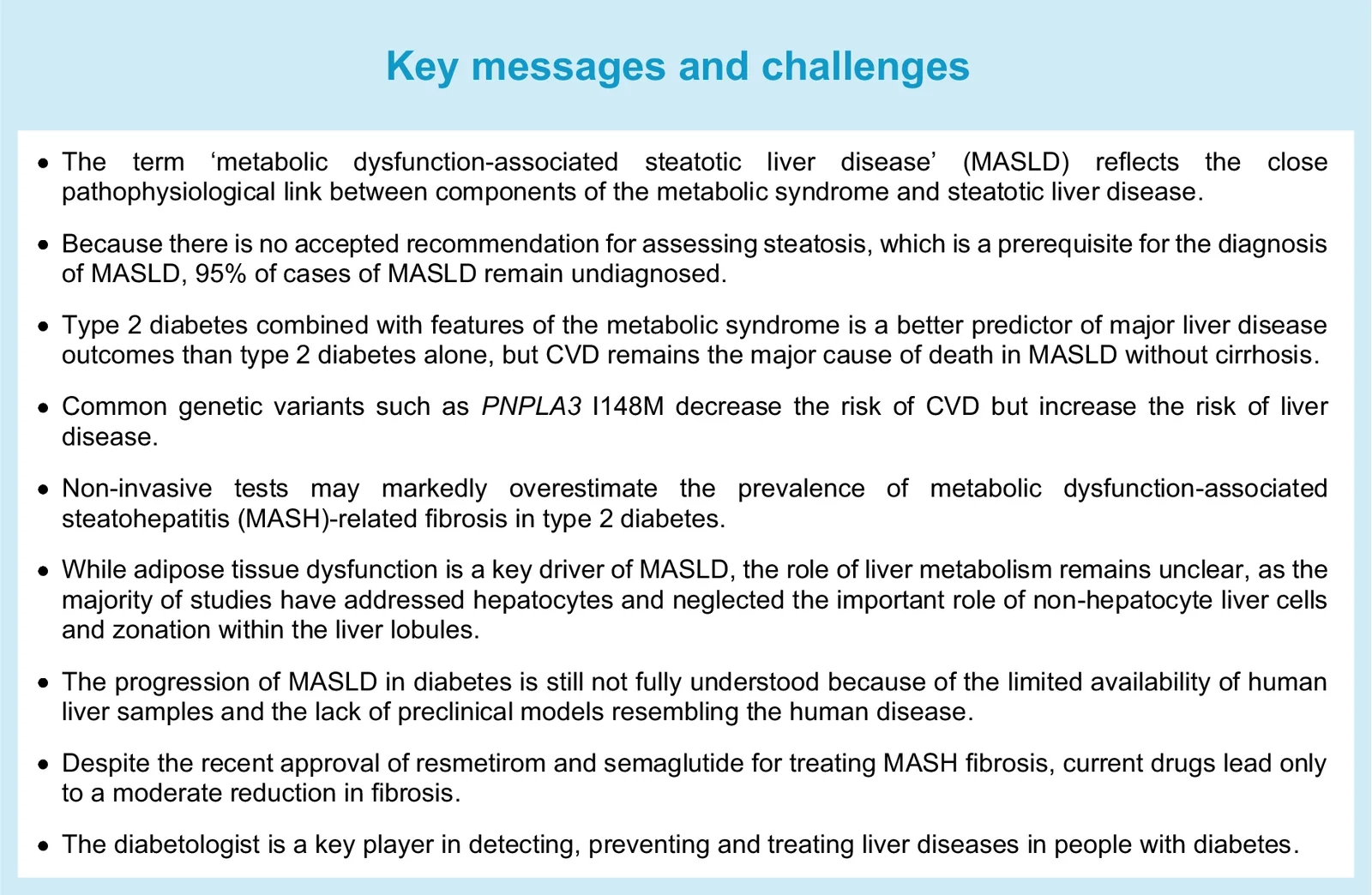

## Introduction

This review focuses on the key features of metabolic dysfunction-associated steatotic liver disease (MASLD), highlighting current challenges in the diagnosis of MASLD and advanced fibrosis in adults with type 2 diabetes. Specific aspects of MASLD in children and young adults are discussed by Segev and Mouzaki [1] in this special issue.

## The inter-relationship between the steatotic liver, type 2 diabetes and CVD

**Fig. 1:**
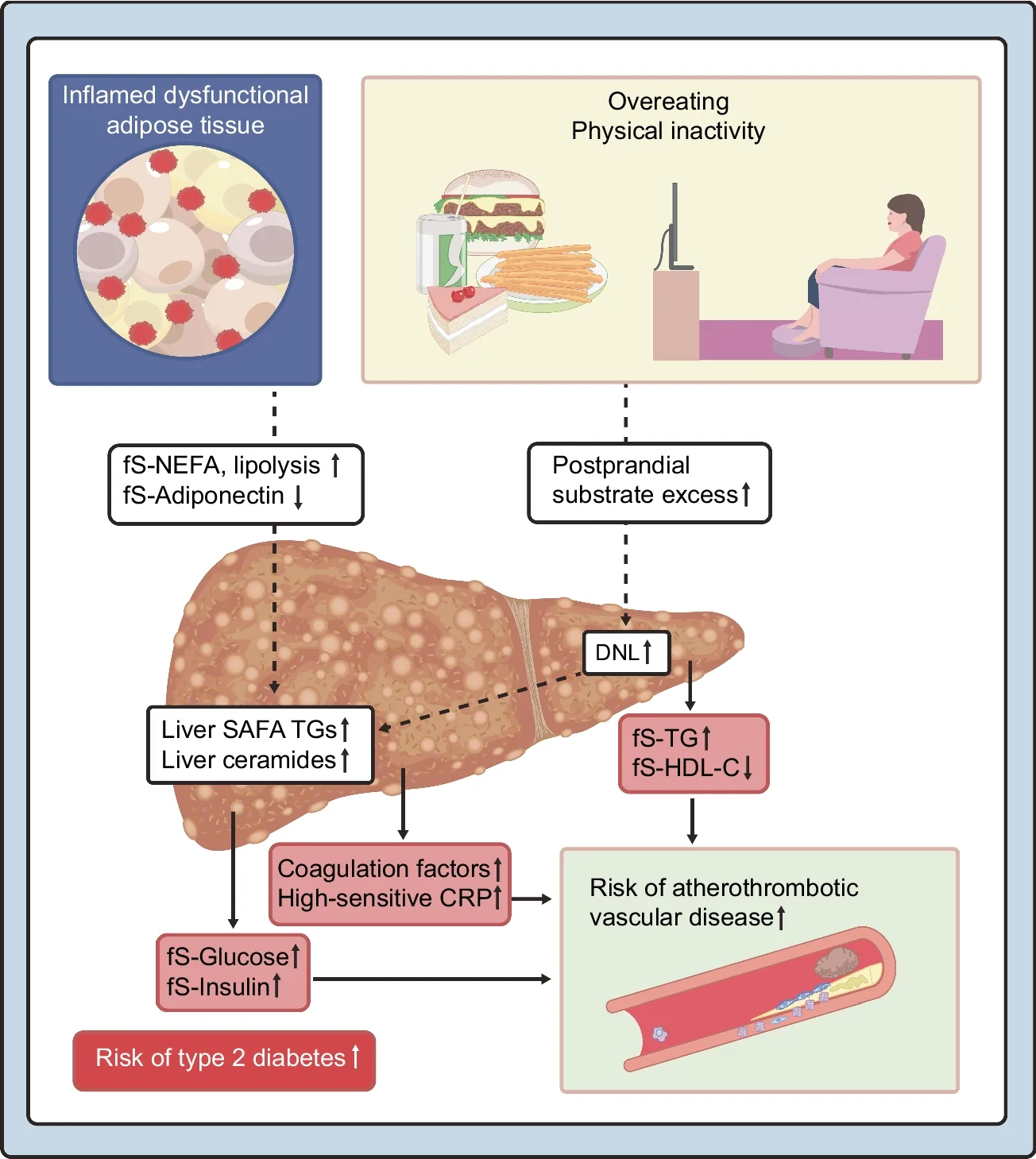
MASLD and the metabolic syndrome: pathogenesis of steatosis. Overeating and inactivity induce postprandial substrate excess, which includes increases in fatty acids, sugars and amino acids. Excess sugars, such as fructose, and amino acids are converted to saturated fatty acids (SAFA) via de novo lipogenesis (DNL) in the liver. Overeating, particularly diets high in SAFAs, also induces inflammation and insulin resistance in adipose tissue, which releases excessive amounts of NEFAs, which are a major substrate for intrahepatocellular triacylglycerol (TG) synthesis in the liver. In individuals with the metabolic syndrome, the liver contains excessive amounts of SAFA-containing TGs and lipotoxic intermediates such as ceramides, which cause insulin resistance and liver damage [8, 12]. The insulin-resistant liver overproduces glucose leading to hyperinsulinaemia and an increased risk of type 2 diabetes, and to hypertriglyceridaemia and low HDL-cholesterol (HDL-C) concentrations. The steatotic insulin-resistant liver also overproduces coagulation factors and CRP, all of which increase cardiovascular risk. This figure is available as part of a downloadable slideset

Since the seminal work of Claude Bernard, the liver has been recognised as the exclusive source of glucose after an overnight fast [2]. In 1951, a group led by the hepatologist Dame Sheila Sherlock used the hepatic venous catheterisation technique to demonstrate that insulin decreases hepatic glucose output and that this action is blunted in people with obesity [3]. Liver biopsies taken from such individuals showed a fatty change, that is, an increase in liver triacyglycerol levels [3]. The association between liver fat and hepatic insulin sensitivity can be dissociated from obesity [4]. The fatty change (steatosis) is characterised by an absolute and relative excess of particularly saturated triacylglycerols [5, 6]. Saturated triacylglycerols can originate from excess intake of dietary saturated fat or excess intake of nutrients such as glucose, fructose and amino acids, which stimulate de novo lipogenesis (Fig. 1) [5–8].

Steatosis is an obesity-independent predictor of type 2 diabetes. This is because hepatic insulin resistance impairs suppression of hepatic glucose production, resulting in hyperglycaemia, which stimulates beta cells to overproduce insulin. The steatotic liver is also resistant to insulin-mediated inhibition of VLDL production, leading to hypertriglyceridaemia, a low HDL-cholesterol concentration and the formation of small dense atherogenic LDL particles [9, 10]. These lipoprotein changes are termed atherogenic dyslipidaemia, which is a key LDL-cholesterol concentration-independent contributor to the risk of CVD in the metabolic syndrome and associated steatosis [11]. Other CVD risk factors overproduced by the steatotic liver include multiple coagulation factors and high-sensitive CRP (Fig. 1) [12].

The steatotic liver thus predisposes to type 2 diabetes and CVD. While steatosis itself could be regarded as a benign condition with respect to liver disease, it may progress to liver damage. The risk is directly proportional to the number of components of the metabolic syndrome that are present. To emphasise this close relationship, in 2023 an expert panel introduced the term ‘MASLD’, which covers a continuum from steatosis to steatohepatitis, and various degrees of fibrosis, culminating in cirrhosis and liver failure, as discussed below [13].

## Diagnostic criteria for MASLD

MASLD is defined as the presence of steatosis in conjunction with at least one of five cardiometabolic risk factors and no other discernible cause [13]. The cardiometabolic risk factors are defined according to the unified criteria for the metabolic syndrome established by multiple international associations and societies [14]. These criteria account for gender or sex differences in the components of the metabolic syndrome. For example, men have more liver triacylglycerol content than women for the same BMI [15, 16] (see Stener-Victorin et al in this special issue for a discussion of MASLD, diabetes and polyendocrine metabolic ovarian syndrome [previously known as polycystic ovary syndrome] across the female life stages [17]). People with MASLD and histologically diagnosed steatohepatitis are designated to have MASH. A liver biopsy is required to diagnose MASH; in addition to steatosis, the diagnosis is based on the presence of lobular inflammation and ballooning degeneration. Fibrosis is classified into four stages, F0-4. The Steatosis, Activity, Fibrosis (SAF) score is widely used for histological assessment of MASLD [18]. Fibrosis in MASH increases as the disease progresses, with fibrosis stages F3 (bridging fibrosis) and F4 (cirrhosis) commonly referred to as ‘advanced fibrosis’.

MASLD and the previously termed ‘non-alcoholic fatty liver disease’ (NAFLD) have very similar clinical profiles and thresholds for non-invasive tests (NITs) and similar long-term outcomes. Published data on NAFLD thus remain applicable to MASLD [19]. As part of the process of renaming NAFLD as MASLD, a separate category called metabolic dysfunction and alcohol-associated liver disease (MetALD) was established as distinct from MASLD (alcohol intake <20 g/day for women and <30 g/day for men). In MetALD, daily alcohol intake is in the range of 20–50 g/day for women and 30–60 g/day for men.

MASLD is an oversimplification of the complex pathogenesis of steatotic liver diseases. As an example, a few common genetic variants explain 30% of the population-attributable risk of steatosis, and the common I148M genetic variant in *PNPLA3* alone explains 10% of liver cirrhosis cases worldwide [20]. The global prevalence of the *PNPLA3* I148M variant is 30–50%. Carriers of the variant have steatosis and an increased risk of steatohepatitis, cirrhosis and hepatocellular carcinoma [21, 22] but a reduced risk of CVD or diabetes [23, 24]. Steatosis due to *PNPLA3* I148M is characterised by antiatherogenic lipid changes, excess polyunsaturated triacylglycerols in the liver and no increase in the production of coagulation factors [25]. Thus, not all forms of MASLD are created equal [26] (see Bilson and Yaghootkar in this special issue for a discussion of the genetic architecture of hepatic steatosis and MASLD in the context of diabetes [27]).

## Why do over 95% of cases of MASLD remain undiagnosed?

### Epidemiological data

In a global meta-analysis, the prevalence of MASLD (2016–2021) was reported to be 69% in people with type 2 diabetes, with ultrasound used as the diagnostic method in 65% of 123 studies [28], which is approximately twofold higher than the prevalence reported in the overall population (34% by ultrasound) [29]. The prevalence of MASH is difficult to determine because diagnosis requires a liver biopsy, which is only performed in highly selected groups of people with steatotic liver disease. The global MASLD burden and the variability in MASLD prevalence among different populations are discussed further by Wild et al [30] in this special issue.

In the population-based NHANES (2017–2023) survey in the USA, the prevalence of advanced fibrosis was estimated non-invasively using vibration-controlled transient elastography (VCTE) with a liver stiffness cut-off of 12 kPa. Using this threshold, the prevalence of advanced fibrosis was reported to be 2.4% [31]. In type 2 diabetes, apparent prevalences of advanced fibrosis averaged 12.2% in a US cohort with obesity [32] and 4.3% in a relatively lean Korean cohort [33] using state-of-the-art magnetic resonance elastography (cut-off 3.63 kPa), and between 10.3% and 22.1% using VCTE (cut-off 9.6–9.7 kPa) [34]. When test sensitivity and specificity are taken into account, the true prevalence of advanced fibrosis in type 2 diabetes is estimated to be <5%, that is, much lower than generally thought (see Qadri [35] in this special issue), indicating that most people with type 2 diabetes do not have fibrosis. Fibrosis, unlike MASH alone [36], predicts mortality in MASLD [23], and the incidence of cardiovascular mortality and heart failure increases with rising fibrosis risk [37]. It is equally important to note that >80% of individuals without cirrhosis (F4) due to MASLD die from non-liver-related causes [38].

A key question for diabetologists is the magnitude of risk for major adverse liver outcomes in individuals with type 2 diabetes or the metabolic syndrome. In a prospective Swedish study, which included 230,992 people with type 2 diabetes without liver disease, the cumulative incidence of major liver disease-related outcomes over 10 years was 1.64% for people with type 2 diabetes and five components of the metabolic syndrome and 0.49% for those with type 2 diabetes alone [39]. The latter risk is low compared with the absolute 10 year risk of CVD of approximately 12% for a 64-year-old European male non-smoker with newly diagnosed type 2 diabetes [40, 41].

MASLD also predisposes to the development of hepatocellular carcinoma (HCC), the most common primary liver cancer. The incidence of HCC is approximately 0.44 per 100 person-years among MASLD cohorts, but reaches 2% per year among those with MASLD cirrhosis [42]. Typically, 80–90% of people with HCC have cirrhosis, but only 50–60% of those with MASLD have cirrhosis at the time of diagnosis of HCC [42]. The presence of impaired glucose tolerance and/or impaired fasting glucose approximately doubles the risk of HCC according to an umbrella review of meta-analyses [43].

### Real-world data

Analysis of health records from four major European territories showed the prevalence of MASLD to be 1.9% in 2014 [44]. This implies that only a small fraction (<5%) of people with MASLD, which has a global prevalence of about 34% in epidemiological studies, are currently diagnosed. In a study of people with an incidental finding of MASLD on imaging (ultrasound, MRI, computed tomography), this diagnosis (ICD10 K76, fatty liver) had only been recorded in 1.6% [45]. These data imply that >95% of cases of MASLD remain undiagnosed. This is understandable because MASLD is generally asymptomatic and there are no inexpensive tools or clinical indications for routine quantification of hepatic steatosis by primary care physicians or diabetologists. Thus, the epidemic of MASLD currently goes largely undetected in clinical practice, which means that many individuals will not receive appropriate preventive or therapeutic measures.

## Should people with type 2 diabetes be screened for liver fibrosis?

The majority of international diabetes guidelines still do not mention MASLD [46]. A consensus report by the ADA [47] and practice guidance from the American Association for the Study of Liver Diseases (AASLD) [48] do recommend screening for liver fibrosis in people with type 2 diabetes. Assessment using the inexpensive fibrosis-4 (FIB-4) index (≈€5 per assessment, if performed only for fibrosis screening, but based on parameters assessed during routing lab testing), which is calculated based on age, AST, ALT and platelet count [48], is recommended every 1–2 years by the AASLD in all those with type 2 diabetes. If ≥1.3, secondary risk assessment using VCTE (≥€100 per assessment) or the serum Enhanced Liver Fibrosis (ELF) test (≥€100 per assessment) is recommended. Individuals with a liver stiffness measurement (LSM) ≥8–10 by VCTE or serum ELF ≥7.7 should be referred to a hepatologist or gastroenterologist, depending on regional availability. The ADA largely recommends the same tests and thresholds for screening in all those with type 2 diabetes and prediabetes, as well as those with obesity and at least one cardiovascular risk factor, every 1–2 years [47].

The above recommendations are well intentioned; however, FIB-4 exceeds 1.3 in approximately 40% of people with type 2 diabetes, all of whom would need to be assessed using VCTE, which is largely available only in gastroenterology and/or hepatology outpatient services. Given that around 61 million people in Europe have type 2 diabetes, this raises concerns about feasibility. Furthermore, given the poor sensitivity and specificity of the tests, 36% of people with advanced fibrosis are missed, despite a true disease prevalence of 5% (18 out of 50 per 1000 people screened), and a huge number (191 out of 1000) are potential candidates to undergo liver biopsy, which could be considered unethical (see Qadri [35]). There is thus an urgent need to revise these strategies. One possibility is to screen only those people with established metabolic syndrome; however, it remains unclear whether, given the available tools, the prevalence of liver disease justifies this approach even in this population. The goal of screening for liver disease is not merely to identify people requiring referral to a gastroenterology/hepatology clinic, but rather to identify those who should undergo maximally effective lifestyle modification and, where appropriate, metabolic surgery to prevent the progression of liver disease. The 2024 EASL-EASD-EASO clinical practice guideline therefore recommends that people with FIB-4 in the range of 1.3–2.67 undergo intensified management of comorbidities before being referred to VCTE [49]. In this context, the diabetologist is well placed to lead management, given their familiarity with therapeutic options for MASLD, including non-pharmacological and pharmacological therapies (see Dufour and Berzigotti [50] and Stefan and Targher [51] in this special issue), as well as bariatric surgery. Of note, the management of people with decompensated cirrhosis should be overseen by a gastroenterologist and/or a hepatologist.

## Novel concepts in MASLD and disease progression

There is broad consensus that the development of MASLD is tightly linked to the pathogenesis of type 2 diabetes [52], with evidence for a specific role of visceral adipose tissue [53]. Figure 1 summarises the key events leading to hepatic steatosis, starting with overnutrition/poor dietary quality and a sedentary lifestyle and leading to adipose tissue dysfunction and altered metabolic fluxes to the liver (see Gastaldelli et al [54] in this special issue). While weight loss can improve these alterations, a better understanding of the pathogenesis of MASLD, and particularly its progression, is needed to address the limitations of current therapies, which reverse steatohepatitis in only a subset of people with MASH and only partially halt or reverse liver fibrosis (see [50, 51]). Specific challenges include: (1) the presence of diabetes subtypes with different risks of MASLD, such as the severe insulin-resistant diabetes subtype, which is associated with increased MASLD incidence and prevalence [55–57]; (2) the identification of liver-specific and cardiometabolic MASLD subtypes, with the latter having a 99% prevalence of type 2 diabetes [58]; and (3) the observation of different responses to MASH therapies in type 2 diabetes [59].

**Fig. 2:**
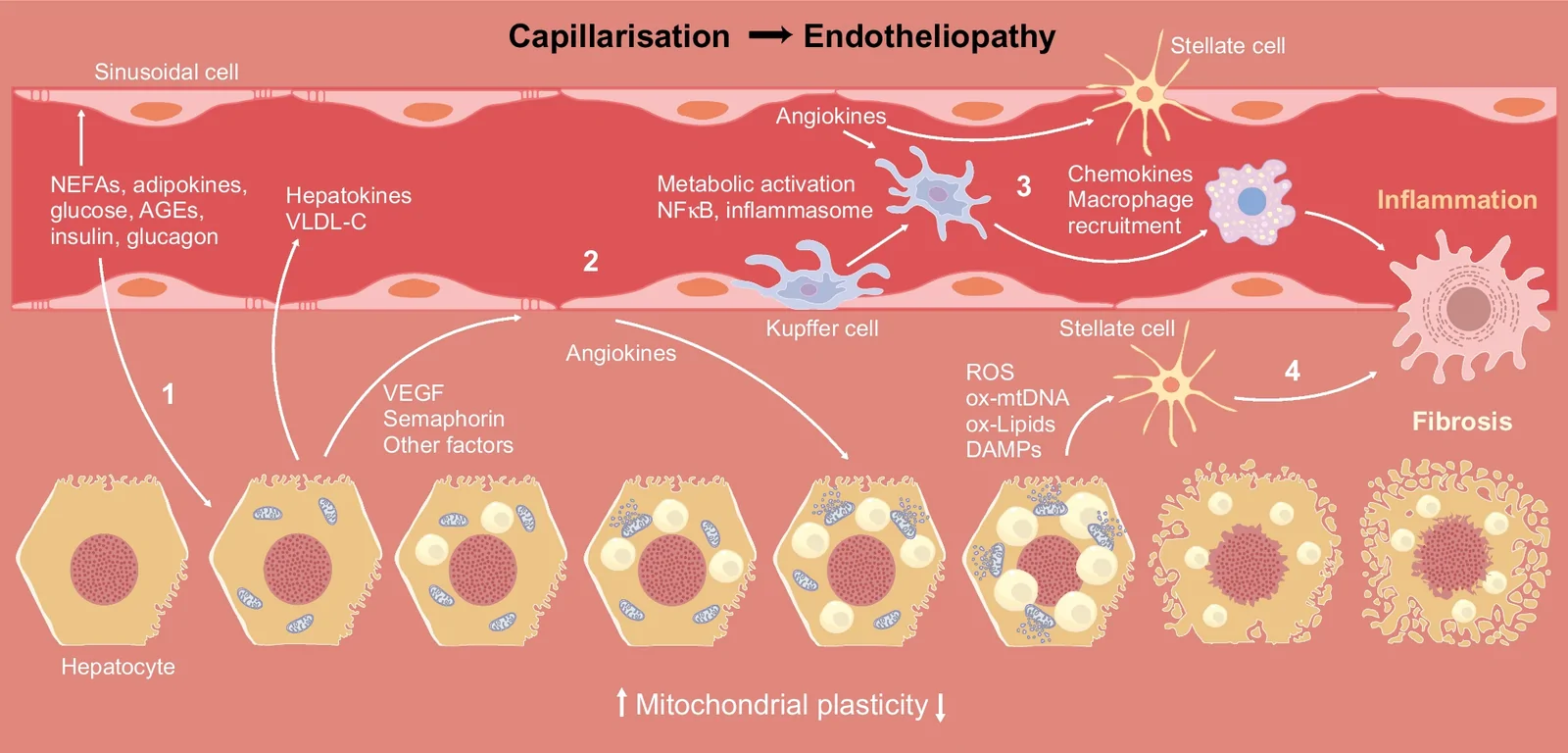
Diabetes mellitus and the liver microenvironment. The figure depicts a schematic of a liver lobule. The time course of development from a healthy liver to steatohepatitis and fibrosis is shown from left to right: (1) metabolic and endocrine factors drive lipid storage and mitochondrial adaptation in hepatocytes; (2) sinusoidal endothelial cells lose their fenestrations and release angiokines, disrupting hepatocellular metabolism and inducing oxidative and ER stress; (3) metabolites and cytokines further activate Kupffer cells, inducing inflammatory pathways; and (4) this ultimately leads to the recruitment of stellate cells and the initiation of fibrosis. DAMPs, damage-associated molecular patterns; mtDNA, mitochondrial DNA; ox, oxidated; ROS, reactive oxygen species; VEGF, vascular endothelial growth factor; VLDL-C, VLDL-cholesterol. This figure is available as part of a downloadable slideset

Over the last decade, studies have focused mostly on whole-liver or hepatocyte metabolism and function, describing lipid-mediated insulin resistance, proinflammatory pathways, endoplasmic reticulum stress [60] and the important role of mitochondrial adaptation to substrate overload (mitochondrial plasticity) [61, 62], which is related to blood glucose concentrations [63] and is lost during MASLD progression, especially in the presence of type 2 diabetes [64] (Fig. 2). Other studies have investigated hepatic fibrogenesis, a process involving interactions between resident and non-resident liver cells that leads to the deposition of extracellular matrix and ultimately to liver failure. These studies have elucidated the shifts in cellular phenotypes and functions that occur, including metabolic changes resembling the Warburg effect in cancer cells [65]. Nevertheless, it remains largely unclear why people with type 2 diabetes progress more rapidly, and more frequently bypass advanced fibrosis stages before developing HCC [66, 67]. However, the application of advanced technologies is now shedding more light on these processes. A single-cell RNA sequencing atlas of paired blood and liver biopsies has identified altered immunoregulatory programmes with MASH progression involving specific populations of regulatory T cells, Kupffer cells, macrophages and dendritic cells [68]. A recent study has highlighted the role of liver sinusoidal cells in the regulation of the crosstalk between hepatocytes, stellate cells, cholangiocytes and immune cells within the lobular microenvironment via angiocrine signalling [69] (Fig. 2). Animal models of obesity, type 2 diabetes and MASLD have demonstrated that saturated fatty acid-induced expression of semaphorin-3A decreases the number of fenestrae through the neuropilin-1/cofilin-1 signalling pathway, which may favour hepatic lipid deposition and VLDL secretion [70]. This and related studies suggest that microangiopathy or endotheliopathy is an early event in the pathogenesis of MASLD in the context of obesity and type 2 diabetes.

Further advances can be expected from three-dimensional liver spheroids, which allow the study of physiological and pathological liver tissue organisation and development. Although hepatocyte organoids can reconstitute the metabolic zonation structure on xenotransplantation in mice [71], they are derived from pluripotent stem cells that only partially recapitulate the human liver. Next-generation human organoids are combined with portal fibroblasts and cholangiocytes from the same donors and form periportal assembloids [72] (Fig. 2). Such models will enable studies on apical–basal cell polarity and, importantly, the zonation of the liver. This will provide a more precise picture of the periportal regulation of gluconeogenesis and of perivenous glycolysis and hypoxic injury, which are important in MASLD [73]. Nevertheless, these approaches should not ignore the relevance of interorgan crosstalk in the pathogenesis and progression of MASLD [74].

## Future directions in MASLD/MASH treatment

Given the primary role of nutrition in the development of MASLD, non-pharmacological interventions to improve the metabolic syndrome, particularly reduction of energy intake and weight loss, have been shown to rapidly resolve liver steatosis, including in type 2 diabetes. Thus, international guidelines and consensus reports recommend lifestyle modification with energy restriction and increased physical activity as a cornerstone of the management of MASLD [47, 49]. However, such interventions are difficult to maintain over longer time periods, even under optimised conditions [75]. Most current clinical studies are limited by challenges in establishing adequate control conditions and achieving long-term sustainability, resulting in only low-grade evidence for specific nutritional and exercise recommendations, except for the recommendation of reduced fructose and alcohol intake in people with MASLD. Of note, innovative strategies such as individualised nutrition-focused telemedicine may lower the incidence of MASLD, MASH and advanced liver disease in people with prediabetes and type 2 diabetes [76]. In addition, surgical weight loss has been demonstrated to be highly effective, even in those with compensated cirrhosis.

While pharmacological therapies exclusively targeting hepatic inflammation or fibrosis [77] have been largely unsuccessful, drugs targeting hepatic or whole-body metabolism, such as the liver-directed thyroid hormone receptor-ß agonist resmetirom, or the glucagon-like peptide-1 receptor agonist semaglutide, were the first to meet the accepted criteria for improving MASH and fibrosis in large Phase III clinical trials (see [51]). Likewise, the therapeutic efficacy of incretin co-agonists and fibroblast growth factor-21 analogues, for which Phase III data are not yet available, also seems to rely mainly on their direct and indirect metabolic effects. This highlights the importance of metabolic medicine and particularly diabetology in the development of innovative MASLD treatments.

A plethora of novel pharmaceutical concepts has moved into clinical development. Table 1 summaries recent Phase I–IIb trials in MASLD/MASH that included (pre)diabetes subgroups and defined a biopsy-based or an accepted NIT-based liver outcome. The data illustrate not only the ongoing interest in intrahepatic lipid metabolism, but also the targeting of bile acid signalling, mitochondrial coupling and inflammatory pathways. In addition to monotherapies, various combination therapies are being investigated, which may offer complementary benefits but also increase the risk of drug interference and adverse effects. Despite some promising results, Table 1 also illustrates the challenges of clinical trials in MASLD. Diagnostic procedures for both MASLD and diabetes are heterogeneous. Likewise, hepatic outcome parameters are still not uniformly defined across studies. This is a result of replacing the liver biopsy, with its inherent risks and limitations, with NITs or indices and imaging approaches, which are not fully standardised or are restricted to specialised centres (see Jonuscheit and Schrauwen-Hinderling [78] in this special issue). Nevertheless, recent developments in non-invasive strategies may be effective not only in detecting liver fibrosis, but also for monitoring therapeutic efficacy in MASH fibrosis [79, 80].

**Table 1:** Summary of recent Phase I–IIb trials on novel drug concepts for MASLD/MASH (2023–2026)

Drug	Phase	Cohort	Cohort size, *n* per group (% diabetes or HbA_1c_ information)	Primary outcome	Findings	Reduction in			Adverse events	Reference
						S	I	F		
FASN inhibitor (denifanstat)	IIb	MASH and F2 or F3 fibrosis	56, 112 (19%, 40%)	≥2 point NAS improvement without fibrosis worsening: common risk difference	Common risk difference (placebo minus treatment): –21%	+	+	+	COVID-19, dry eye, alopecia	[91]
				Steatohepatitis resolution without fibrosis worsening	Common risk difference (placebo minus treatment): −13%					
DGAT2 inhibitor (ION224)	II	MASH and F1–F3 fibrosis	23–46 (38–54%)	≥2-point NAS reduction + ≥1-point improvement in ballooning or lobular inflammation, without fibrosis worsening at week 51	Risk difference (placebo minus treatment): –27% (90 mg); −40% (120 mg)	+	(+)	(+)		[92]
DGAT2 inhibitor (ervogastat) monotherapy and with ACC inhibitor (clesacostat)	II	MASH and F2 or F3 fibrosis	30–48 (52–74%)	Proportion with MASH resolution without fibrosis worsening, ≥1 stage fibrosis improvement without MASH worsening, or both, at week 48	Difference from placebo in proportion achieving endpoint: NS (monotherapy); 0.25, 0.27 (combination therapies)	+	+	(+)	Combination therapy: increased lipids and apolipoproteins and worsened diabetes	[93]
Liver-targeted FFAR 1/4a (icosabutate)	IIb	MASH and F1–F3 fibrosis	62–67 (42–49%)	Proportion with MASH resolution with no fibrosis worsening at week 53 in the 600 mg arm	NS	+	(+)	(+)		[94]
RNA-interfering agent targeting *HSD17B13* mRNA (rapirosiran)	I	Study A: healthy adults; study B: MASH	A: 58 (HbA_1c_ NR) B: 46 (HbA_1c_ increased)	Safety, kinetics		–^a^	+	+	Injection site reaction	[95]
Intrahepatic activated mitochondrial uncoupler 2,4-DNP (HU6)	IIa	MASLD BMI 28–45 kg/m^2^	19–21 (borderline HbA_1c_)	Relative change in MRI-PDFF from baseline to day 61	–27% (150 mg); −36% (300 mg); −33% (450 mg); 5.4% (placebo)	+	NR	NR	Flushing, diarrhoea, palpitations	[96]
ACLY inhibitor (enedioic acid analogue 326E)	Ib/IIa	MASH BMI >25 kg/m^2^	9–12 (8–22%)	Safety, kinetics		+^b^	NR	NR		[97]
TERN-501 (THRβa) monotherapy and with FXRa (TERN-101)	IIa	MASH?	22–24 (27–57%)	Relative change in MR-PDFF from baseline at week 12	–28% (3 mg); −45% (6 mg); −21%, −48% (combination therapies); −4% (placebo)	+^c^	(+)^c^	(+)^c^	Pruritus, diarrhoea, free thyroxine decreases at week 2 and week 6	[98]
Berberine ursodeoxycholate (HTD1801)	II	MASH?	33–34 (increased HbA_1c_)	Composite response of ≥2-point NAS improvement + MASH resolution	52% vs 24% placebo	+^d^	(+)^d^	(+)^d^	Diarrhoea, nausea	[99]
Monoclonal antibody, activating FGFR1c/β-klotho co-receptor complex (MK-3655)	IIb	MASH and F2 or F3 fibrosis	44–48 (49–55%)	MASH resolution without fibrosis worsening at week 52	NS	+/–	–	–	Diarrhoea	[100]
LTA4Hi (LYS006) monotherapy and with non-bile acid FXR agonist (tropifexor)	II	MASH?	20, 21 (increased HbA_1c_)	Safety, kinetics		(+)^e^	NR	NR	Combination therapy: pruritus, LDL-cholesterol increase, HDL-cholesterol decrease	[101]
Liver-targeted antisense oligonucleotide against *PNPLA3* mRNA (AZD2693)	I	MASH? *PNPLA3* 148M+	46–16 (4–67%)	Safety, kinetics		+^f^	NR	NR	Transient ALT and AST increases	[102]
Low-dose acetylsalicylic acid	II	MASLD/ MASH and F0–F3 fibrosis	40, 40 (38–40%)	Mean absolute change from baseline in liver lipids at month 6 measured using ^1^H-MRS	−6.6% vs +3.6% placebo (∆−10%)	+	NR	NR		[103]

Diagnostic method: ^a^MRI-PDFF; ^b^MRI-PDFF at week 4; ^c^cT1; ^d^MRI-PDFF and/or cT1; ^e^MRI-PDFF (no placebo control); ^f^MRI-PDFF at week 12

+, significant improvement; (+) non-significant trend towards improvement; –, no improvement; a, agonist; ACC, acetyl coenzyme A carboxylase; ACLY, ATP-citrate lyase; DGAT, diacylglycerol O-acyltransferase; DNP, dinitrophenol; F, fibrosis; FASN, fatty acid synthase, FFAR, free fatty acid receptor; FGFR, fibroblast growth factor receptor; FXR, farnesoid X receptor; ^1^H-MRS, proton magnetic resonance spectroscopy; HSD, hydroxysteroid 17-beta dehydrogenase; I, inflammation; LTA4H, leukotriene A4 hydrolase; MRI-PDFF, MRI-protein density fat fraction; NAS, NAFLD Activity Score; NR, not reported; NS, not significant; PNPLA 3, patatin-like phospholipase domain-containing protein 3; S, steatosis; THR, thyroid hormone receptor

The majority of clinical trials on MASLD do not or are not able to address the predominant causes of MASLD-related morbidity and mortality, particularly in people with type 2 diabetes and metabolic cardiovascular renal disease [81]. Although a causal relationship between MASLD and CVD is still under debate, people with MASLD mostly die from CVD (see Kahl et al [82] in this special issue). As discussed by Qadri [35], the 10 year cumulative incidence (*n*=406,770, >21 million person-years of follow-up) of major adverse liver outcomes is 1.8% in people with type 2 diabetes without other components of the metabolic syndrome than hyperglycaemia, 3% in those with type 2 diabetes with all components of the metabolic syndrome, and 0.8% in healthy individuals [41]. In contrast, in people with recently diagnosed type 2 diabetes and a similar CVD risk burden, the 5 year cumulative incidence of major CVD approaches 30% [83]. Recently, a pragmatic roadmap has been proposed for the design of integrated multiorgan trials, outlining enriched trial populations and shared non-invasive surrogate outcomes, with the ultimate aim of establishing platform trials using master protocols in both cardiorenal and MASLD research [84].

## Take-home messages and future research gaps

The slow progression of MASLD, combined with the focus on classical diabetes complications in most diabetes guidelines, is a major contributor to the lack of awareness surrounding its diagnosis. Thus, there is an immediate need to recognise MASLD as a diabetes comorbidity, and the management of liver disease should be included in all diabetes guidelines.

Despite the overall efficacy of NITs, their diagnostic precision remains suboptimal, calling for further analyses of large consortia for biomarker discovery, such as LIVERAIM (https://www.liveraim.eu/ [2025]), NIMBLE [85] and LITMUS [86]. Such analyses would benefit from the inclusion of cohorts of comprehensively phenotyped individuals with type 2 diabetes to improve future risk stratification tools. This would also help to address the unmet need to discriminate reliably between slowly and rapidly progressing liver disease and to predict treatment responses.

Better understanding of disease progression calls for refined approaches to generate functional organoids derived from human liver samples along with single-cell and spatial multi-omics. Further integration of immune cells and vascular cells into assembloids [72] would allow the recreation of the physiological microenvironment, thereby facilitating the detection of novel therapeutic targets. Furthermore, given the limitations of preclinical diabetes–MASH fibrosis models, novel in vitro models for high-throughput drug screening could accelerate the validation of therapeutic concepts. Such models [87] could improve drug-testing strategies and prospectively reduce the number of animal studies and expensive, time-consuming—and frequently disappointing—clinical trials. Furthermore, integration of multi-omics data and AI-driven analyses, as already demonstrated in digital pathology [88], are essential for gaining further insights into obesity- and type 2 diabetes-related subgroups with excessive risks of MASLD progression and to improve precision diabetology through refined stratification [57].

Nevertheless, all these activities must ultimately focus on patient-centred, precise multidisciplinary management of MASLD in the context of obesity and diabetes. This starts with reducing stigma and discrimination against individuals with MASLD and fostering interaction among all stakeholders as outlined in the People-First Liver Charter, which has been endorsed by many scientific associations and journals [89]. To achieve the goal of doubling the diagnostic rate of at-risk MASH will rely on a paradigm shift, including greater awareness among clinicians and expansion of community-based diagnostic capabilities along with in-depth analyses of cost-effective models of prevention and treatment [90].

## Supplementary Information

Below is the link to the electronic supplementary material.

Slideset of figures (PPTX 535 KB)

## Abbreviations

CRP: C-reactive protein
ELF test: Enhanced Liver Fibrosis test
FIB-4 index: Fibrosis-4 index
HCC: Hepatocellular carcinoma
MASH: Metabolic dysfunction-associated steatohepatitis
MASLD: Metabolic dysfunction-associated steatotic liver disease
MetALD: Metabolic dysfunction and alcohol-associated liver disease
NAFLD: Non-alcoholic fatty liver disease
NIT: Non-invasive test
PNPLA3: Patatin-like phospholipase domain-containing protein 3
SAF score: Steatosis Activity Fibrosis score
VCTE: Vibration-controlled transient elastography
